# Unraveling the Structural and Compositional Peculiarities in CTAB-Templated CeO_2_-ZrO_2_-MnO_x_ Catalysts for Soot and CO Oxidation

**DOI:** 10.3390/nano13243108

**Published:** 2023-12-09

**Authors:** Maria V. Grabchenko, Natalia N. Mikheeva, Grigory V. Mamontov, Vicente Cortés Corberán, Kseniya A. Litvintseva, Valery A. Svetlichnyi, Olga V. Vodyankina, Mikhail A. Salaev

**Affiliations:** 1 Laboratory of Catalytic Research, Tomsk State University, 634050 Tomsk, Russia; marygra@mail.ru; 2 Research Laboratory of Porous Materials and Sorption, Tomsk State University, 634050 Tomsk, Russiavodyankina_o@mail.ru (O.V.V.); 3 Instituto de Catálisis y Petroleoquímica (ICP), Consejo Superior de Investigaciones Científicas (CSIC), 28049 Madrid, Spain; vcortes@icp.csic.es; 4 Boreskov Institute of Catalysis SB RAS (BIC SB RAS), 630090 Novosibirsk, Russia; 5 Department of Physics, Novosibirsk State University, 630090 Novosibirsk, Russia; 6 Laboratory of Advanced Materials and Technology, Siberian Physical Technical Institute, Tomsk State University, 634050 Tomsk, Russia

**Keywords:** CeO_2_, MnO_x_, ternary oxide catalysts, soot combustion, CO oxidation

## Abstract

Structure–performance relationships in functional catalysts allow for controlling their performance in a wide range of reaction conditions. Here, the structural and compositional peculiarities in CTAB-templated CeO_2_-ZrO_2_-MnO_x_ catalysts prepared by co-precipitation of precursors and their catalytic behavior in CO oxidation and soot combustion are discussed. A complex of physical–chemical methods (low-temperature N_2_ sorption, XRD, TPR-H_2_, Raman, HR TEM, XPS) is used to elucidate the features of the formation of interphase boundaries, joint phases, and defects in multicomponent oxide systems. The addition of Mn and/or Zr dopant to ceria is shown to improve its performance in both reactions. Binary Ce-Mn catalysts demonstrate enhanced performance closely followed by the ternary oxide catalysts, which is due the formation of several types of active sites, namely, highly dispersed MnO_x_ species, oxide–oxide interfaces, and oxygen vacancies that can act individually and/or synergistically.

## 1. Introduction

Catalytic technologies are widely implemented in both large-scale chemical manufacturing and solving environmental challenges. The latter is linked to the reduction of the impact of various hazardous substances (CO, particulate matter, VOCs) on human health and the environment [1,2]. Over the last decades, a number of catalyst formulations have been introduced for the oxidation of CO and soot, and such catalysts are mostly based on noble metals and transition metal oxides. The noble metal-based supported catalysts utilizing Rh, Pt, or Pd were widely studied due to their high activity in total oxidation reactions [3,4,5,6]. However, CO was found strongly adsorbed on such metals, resulting in a significant reduction in active oxygen content that suppressed the low-temperature oxidation activity and the need to apply elevated temperatures [7]. Moreover, these metals feature rather high costs and limited availability. This motivated a search for alternative less expensive and abundant catalyst components that demonstrate similar or superior performance in said reactions.

A promising alternative comprises the catalysts based on oxides of transition metals (pristine and mixed oxides [8,9], spinels [10,11,12]). These materials combine a number of advantages, including availability, thermal stability, improved service period, resistance to catalytic poisons, etc. Among such materials, ceria brings about high research interest [13] since it demonstrates enhanced abilities to accumulate and release oxygen, and features rather low costs. To further boost CeO_2_ performance in terms of improved oxygen mobility and capacity as well as mechanical characteristics, the interactions of ceria with other oxides, i.e., ZrO_2_, MnO_x_, SnO_2_, are considered in binary and ternary formulations [14,15,16,17,18,19,20]. In a number of cases, the ternary mixed oxide systems exhibited rather high performance, while the reasons for such a behavior remain under debate.

Templating methods allow for creating oxide-based catalysts with improved structural and performance properties [21]. A key focus to create porous oxide catalysts using template methods is a high specific surface area that ensures effective mass transfer and diffusion of reagents to the active sites of the catalyst. The size, shape, and distribution of pores in the catalyst can be controlled by choosing the type of template and synthesis conditions [22,23,24]. Among the templates to synthesize the oxide catalysts [25,26], the cationic surfactant cetyltrimethylammonium bromide (CTAB) is actively used [27,28] due to the formation of micelles that help the precipitation of the oxides in the form of nanoparticles. The CTAB also allows for stabilizing the nanoparticles, preventing them from agglomeration.

Recently, several attempts were undertaken to show the effects of template-based preparation methods on the structure of ternary CeO_2_-ZrO_2_-MnO_x_ oxides and their performance in the complete oxidation of CO and soot. Thus, the CeO_2_-ZrO_2_ prepared with a sawdust template showed improved performance in CO oxidation, which was attributed to higher structural defectiveness [29]. The soot combustion activity was rather low, while the CTAB-templated sample showed the opposite behavior. In Ref. [30], for the sample prepared using the evaporation-induced self-assembly method, the Mn^2+^/Mn^3+^ ions were incorporated into the CeO_2_-ZrO_2_ lattice to form the MnO_x_ particles evenly distributed over the surface and bulk of the sample. The activity of such a sample was lower as compared to the one prepared by the MnO_x_ impregnation over the mixed oxide support, where only local surface areas contained the MnO_x_ species to ensure additional adsorption sites. With that, it is not clear how the performance of such ternary catalysts can change if the Mn-related component is introduced simultaneously with other catalyst components.

To the best of our knowledge, there have been no attempts to prepare ternary CeO_2_-ZrO_2_-MnO_x_ catalysts using CTAB-templated synthesis when the precursor of the MnO_x_ component is introduced not by impregnation of the CeO_2_-ZrO_2_ support, but simultaneously with other oxide precursors. Despite thorough investigation, there are open questions related to the nature and function of the surface defects. A landscape of catalyst formulations that can demonstrate the synergistic action reflected in the enhanced catalyst performance can also be widened.

The present work is focused on the structural and compositional peculiarities in CTAB-templated CeO_2_-ZrO_2_-MnO_x_ catalyst and its catalytic behavior in both CO oxidation and soot combustion. A complex of physical–chemical methods (low-temperature N_2_ sorption, XRD, TPR-H_2_, Raman, XPS) is used to elucidate the features of the formation of interphase boundaries in multicomponent oxide systems and the defects formed.

## 2. Materials and Methods

### 2.1. Synthesis of CeO_2_-ZrO_2_-MnO_x_ Oxide Systems

To prepare the oxide-based systems, a hydrothermal synthesis with CTAB as a structure-forming additive was used [31]. The oxide precursors (Ce(NO_3_)_3_·6H_2_O, Mn(NO_3_)_2_·nH_2_O, ZrO(NO_3_)_2_·nH_2_O) were dissolved in water, and the CTAB was added to the obtained solution with a molar ratio of CTAB to oxides of 1:3. Then, the mixture was put into an autoclave at a temperature of 100 °C for 24 h. After filtering, the samples were dried at 80 °C for 16 h and then calcined at 600 °C for 2 h. The Ce/Mn and Ce/Zr ratios were selected to be no less than 4 and 1.5, respectively, based on the literature data [32,33]. The sample designations show the nominal chemical composition (Table 1).

### 2.2. Materials Characterization

A complex of physical–chemical methods was used to characterize the prepared samples to provide comprehensive information about their composition, structure, and properties.

The catalysts were characterized by low-temperature nitrogen adsorption to measure specific surface area, porosity, and pore size distribution (“3Flex”, Micromeritics, Norcross, GA, USA). The samples were previously degassed under vacuum (200 °C, 2 h). The surface area and pore size distributions were determined using the BET and BJH methods, respectively.

X-ray fluorescence analysis (XRF) was used to determine the elemental composition of the obtained samples (XRF-1800, Shimadzu, Tokyo, Japan). The applied voltage, current, and diaphragm were 40 kV, 95 mA, and 10 mm, respectively. The Rh-based anode was used as a source.

X-ray phase analysis (XRD) was used to study the phase composition of samples, estimate the size of crystallites of components, and calculate the structural parameters (XRD-7000, Shimadzu, Tokyo, Japan). The range of 2θ was 10–70. CuKα radiation was used. The crystalline phase composition was established using the PDF database. The POWDER CELL 2.5 software package was used to determine the crystal cell indexing, starting from powder diffraction data with the Lorentz simulation. The crystal size and microstrains were calculated using the Williamson–Hall method.

Temperature-programmed reduction in hydrogen (TPR-H_2_) was used to study the features of the reduction of active components in synthesized samples (“Autochem 2950”, Micromeritics, Tokyo, Japan). The temperature range was 25–900 °C. The device was equipped with a thermal conductivity detector. A mixture of 10 %vol. H_2_ in argon was used. The absence of water in the gas mixture was controlled by a trap with a mixture of isopropanol and liquid nitrogen with temperature of −86 °C. The heating rate and gas flow rate were 10 °C/min and 20 mL/min, respectively. The air pretreatment in the temperature-programmed oxidation mode (20 mL/min) was carried out at temperatures up to 500 °C with an exposure time of 20 min.

Raman spectroscopy was used to confirm the defective structure of as-prepared samples (InVia confocal Raman dispersive spectrometer, Renishaw, Wharton, UK). The Leica microspore (50× objective) was employed. The excitation was ensured by a solid-state Nd:YAG laser. The wavelength, radiation power, and spectral resolution were 532 nm, 100 mW, and 2 cm^−1^, respectively. The powder samples were analyzed.

X-ray photoelectron spectroscopy (XPS) was used to determine the chemical state of active components (SPECS Surface Nano Analysis GmbH, Berlin, Germany). The PHOIBOS-150-MCD-9 hemispherical analyzer and the UXC 1000 X-ray source with a double Al/Mg anode were applied. Non-monochromatized Al Kα radiation was used. The charging effect was accounted for by the u‴ peak with a binding energy of 916.7 eV in the Ce3d spectrum. The relative concentrations of elements in the analysis zone were determined from the integral intensities of the XPS spectra to take the photoionization cross-section into account. To subtract the background, the Shirley method was used. The data were processed using CasaXPS software. The peak approximation involved the asymmetric LF function for the Mn2p spectrum and a symmetric function summing the Gauss and Lorentz functions for other elements.

### 2.3. Catalytic Tests in Oxidation CO and Soot

CO oxidation tests were carried out in a tubular U-shaped quartz reactor (i.d. = 6 mm) at atmospheric pressure in the temperature-programmed heating mode (“AutoChem”, Micromeritics, Norcross, GA, USA). The concentrations of CO (*m*/*z* = 28), CO_2_ (*m*/*z* = 44), and O_2_ (*m*/*z* = 32) were controlled using the gas mass spectrometer UGA-300 (Stanford Research Systems, Sunnyvale, CA, USA). The sample pretreatment was carried out using a H_2_ flow (up to 300 °C, 1 h). The particle fraction was 0.25–0.5 mm. The reaction mixture comprised 1 vol% CO, 5 vol% O_2_, and 94 vol% He. The sample mass, the flow rate, and the heating rate were 0.1 g, 100 mL/min, and 10 °C/min, respectively.

Soot combustion activity was estimated using synchronous thermal analysis (STA 449F1 thermogravimeter, NETZSCH, Selb, Germany). Carbon black (Micromeritics, Norcross, GA, USA) was used, and 5 wt% soot was mixed with the catalyst. Tight contact mode was ensured by mixing the components in an agate mortar for 10 min. The temperature range was 100–800 °C. The heating rate was 10 °C/min in an air flow.

## 3. Results

### 3.1. Chemical Composition

Real catalyst compositions were determined using the XRF method. The values obtained were similar to the nominal ones. No other impurity elements were detected either by XRF or XPS.

The analysis of the Ce/Mn and Ce/Zr ratios determined based on the XRF and XPS data (Table 1) shows that the Ce/Mn (XPS) ratio for Ce_0.9_Mn_0.1_O_2_ and Ce_0.5_Mn_0.2_Zr_0.3_ samples are lower than those determined by XRF, indicating a surface enrichment in Mn. The contrary is observed in Ce_0.5_Mn_0.3_Zr_0.2_O_2_, whose surface is impoverished in Mn, probably due to the effect of the surface Zr. The Ce/Zr (XPS) ratio is lower for Ce_0.6_Zr_0.4_O_2_ and Ce_0.5_Mn_0.2_Zr_0.3_O_2_ samples, implying a surface enrichment with Zr. This can be linked to the effect of calcination temperature [34].

### 3.2. Low-Temperature Nitrogen Adsorption

**Figure 1:**
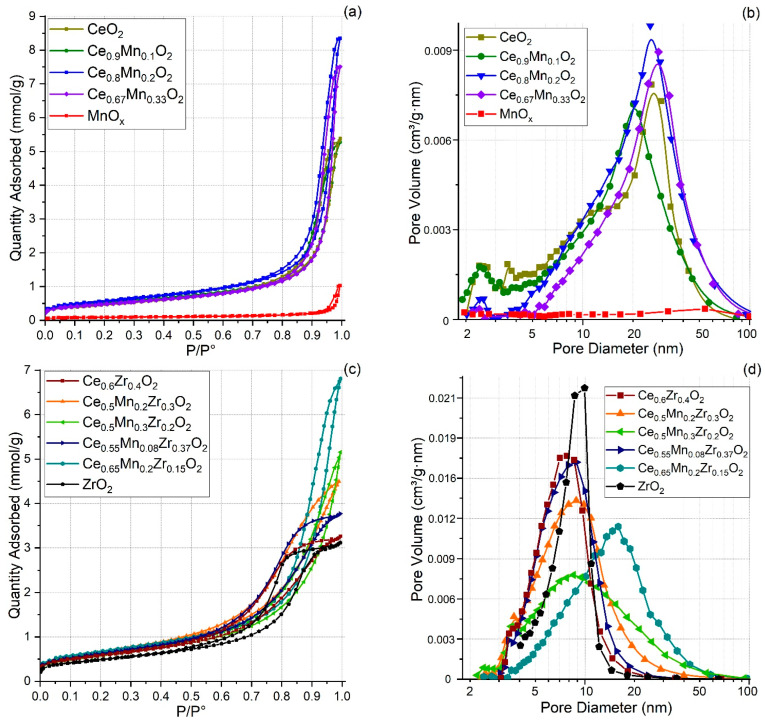
(**a**,**c**) Isotherms of N_2_ adsorption–desorption for the obtained samples; (**b**,**d**) Pore size distributions.

Table 2 shows the specific surface areas (SSAs), average pore sizes, and pore volumes measured by N_2_ adsorption–desorption. The isotherm for CeO_2_ (Figure 1a) is characterized by a hysteresis loop with the H3 type, which indicates the formation of lamellar particles [35]. A wide pore size distribution is observed. For the ZrO_2_ sample, the H2 type hysteresis loop is observed (Figure 1c), which indicates a relatively wide size distribution of pore cavities compared to the one of the necks. The pore size distribution is fairly narrow. For the MnO_x_ sample, the H3 type hysteresis loop is observed [35], with a rather low SSA and a small pore volume being observed. Due to these characteristics, the pore size is rather difficult to determine for this sample.

Binary oxide systems consisting of CeMnO_x_ are characterized by a hysteresis loop similar in shape to the one for CeO_2_ (Figure 1a,b), as well as a fairly wide distribution of pore sizes and a mean-specific surface area. The Ce_0.9_Mn_0.1_O_x_ sample is characterized by a pore size distribution with small and large pores with sizes of 2–4 nm and 5–50 nm, respectively (Figure 1d). The Ce_0.6_Zr_0.4_O_2_ sample is characterized by a hysteresis loop similar in shape to the one for zirconium oxide.

Ternary oxide systems are characterized by an adsorption–desorption isotherm with the H2 type hysteresis loop (Figure 1c). A narrow distribution of pore diameters (3–20 nm, except for the Ce_0.65_Mn_0.2_Zr_0.15_O_2_ sample featuring distribution of 3–50 nm) is observed with a slight shift in the maximum of the pore size distribution (up to 9 nm) and a significant decrease in the pore volume (Figure 1d) [34]. It is noteworthy that the maximum of the distribution of the above samples is similar to the one for the Ce_0.6_Zr_0.4_O_2_ sample. This fact may indicate a strong interaction of oxides at the preparation stage with the preservation of the CeO_2_-ZrO_2_ structure during the incorporation of Mn^n+^ ions into the porous space of CeO_2_-ZrO_2_. In the case of the Ce_0.65_Mn_0.2_Zr_0.15_O_2_ sample (which the Mn/Ce+Zr ratio is the same as in the Ce_0.5_Mn_0.2_Zr_0.3_O_2_ sample but has a lower Zr/Ce ratio), the maximum of the pore size distribution is shifted to 18 nm. This may indicate that a low Zr/Ce ratio does not allow Mn to be uniformly distributed in the CeO_2_ and CeO_2_-ZrO_2_ structures.

### 3.3. X-ray Phase Analysis (XRD)

**Figure 2:**
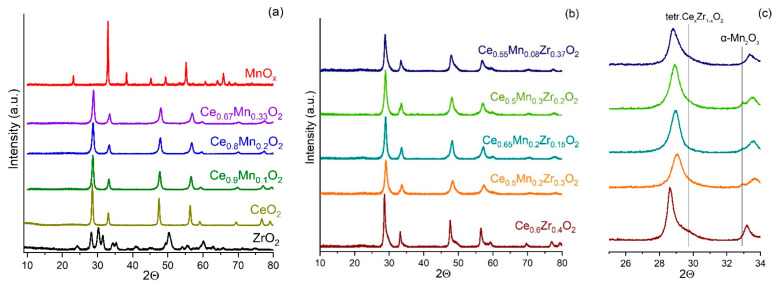
XRD patterns for the prepared samples: (**a**) individual and Ce-Mn oxides, (**b**) Ce-Zr and ternary oxides, (**c**) excerpt from patterns (**b**).

The phase composition of the obtained samples was studied by the XRD and analyzed by Rietveld refinement using the POWDER CELL 2.5 package program. The goodness of the fit was controlled using the low R_p_ and R_wp_ values. The lattice parameters (*a*, *b*, and *c*), microstrain value (∆d/d), and crystalline size (D_XRD_) were calculated using the Williamson–Hall method [36] (Table 3). Figure 2 shows the XRD patterns.

The synthesized manganese oxide is characterized by the Mn_2_O_3_ cubic bixbyite phase (ICSD #76087), with a particle size of 43 nm (Figure 2, Table 3). The pristine ceria is formed by the fluorite CeO_2_ phase (ICSD #28753) with a crystallite size of 28 nm. The individual ZrO_2_ is characterized by the reflections related to both monoclinic (ICSD #18190) (59%) and tetragonal phases (ICSD #51051) (41%), both with the crystallite size of 15 nm.

Binary systems consisting of cerium and manganese oxides are characterized by the presence of reflections of cubic cerium oxide (Figure 2a). However, the reflections shift to higher angles with an increase in the content of manganese oxide, which indicates the substitution of Ce^4+^ ions (i.r. = 0.88 Å) by those of manganese Mn^n+^ (i.r. 0.81 Å, 0.72 Å, and 0.52 Å for Mn^2+^, Mn^3+^, and Mn^4+^, respectively) in the CeO_2_ network with the formation of a solid solution with a fluorite-type (F-type) structure. According to the Vegard’s law, the compositions of the solid solutions were calculated as Ce_0.94_Mn_0.06_O_2−δ,_ Ce_0.9_Mn_0.1_O_2−δ_, and Ce_0.89_Mn_0.11_O_2−δ_ for Ce_0.9_Mn_0.1_O_2_, Ce_0.8_Mn_0.2_O_2_, and Ce_0.67_Mn_0.33_O_2_, respectively. These calculated compositions of the solid solutions indicate that only a small part of manganese is included into the structure, and the rest is segregated in the form of highly dispersed Mn_2_O_3_ and/or Mn_3_O_4_.

The Ce_0.6_Zr_0.4_O_2_ sample is characterized by the presence of reflections from the CeO_2_ phase and a shoulder from the cubic zirconia phase (ICSD #53998) (Figure 2b). The Rietveld refinement of the Ce_0.6_Zr_0.4_O_2_ powder pattern reveals defective Ce_1−x_Zr_x_O_2−δ_ (47%) and Ce_x_Zr_1−x_O_2−δ_ (53%) solid solutions with a cubic structure. However, the formation of the Ce_x_Zr_1−x_O_2_ solid solution with a tetragonal structure cannot be ruled out due to the comparable R_p_ and R_wp_ values. The solid solution compositions evaluated according to Vegard’s law are Ce_0.92_Zr_0.08_O_2−δ_ (F-type) and Ce_0.41_Zr_0.59_O_2−δ_ (F-type) or Ce_0.18_ Zr_0.82_O_2−δ_ (tetragonal type). For Ce_x_Zr_1−x_O_2_ with 0.3 ≤ x ≤ 0.65, the formation of the most stable tetragonal phase was shown (see Ref. [37] and references within).

**Figure 3:**
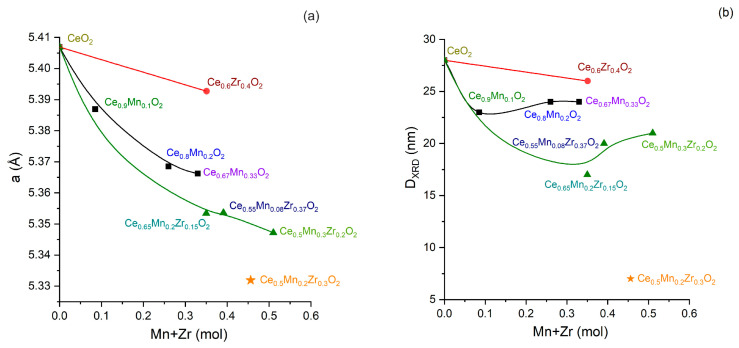
Relationships between cell parameter of CeO_2_ (**a**) and crystallite size (**b**) and fraction of substitution metals.

Ternary oxide systems are characterized by the reflections of the cubic ceria with a broad shoulder at ~30° and a small one at 32.9°, which can be associated with the contribution of the zirconia phase and partial segregation of the α-Mn_2_O_3_ (ICSD #9090) on the surface, respectively (Figure 2b,c). The significant decrease in the lattice parameter of the CeO_2_ phase for the CeO_2_-ZrO_2_-MnO_x_ ternary systems relative to the reference samples of CeO_2_, CeO_2_-MnO_x_, and CeO_2_-ZrO_2_ (See Figure 3a) is due to the introduction of smaller Mn^3+^ and Mn^4+^ ions (i.r. are 0.70 and 0.52 Å, respectively) and Zr^4+^ (i.r. is 0.82 Å) compared to the Ce^4+^ ion (i.r. is 0.88 Å) to form the solid solutions. The Ce_0.5_Mn_0.2_Zr_0.3_O_2_ sample is characterized by the lowest lattice parameter (5.331 Å), which can indicate the highest amount of Mn and Zr ions incorporated into the CeO_2_ structure.

The Ce^4+^ substitution hinders the crystallite growth for the CeO_2_-ZrO_2_-MnO_x_ samples (in the range of 7–21 nm), and this results in a microstrain increase (Δd/d = 0.0043–0.0094) due to the additional number of oxygen-anion vacancies formed in the Ce_1−(x+y)_Zr_x_Mn_y_O_2−δ_ solid solutions. When Mn dopant is added to CeO_2_, the crystallite size decreases slightly (see Figure 3b). Moreover, upon further increase in the Mn/Ce ratio, the crystallite size remains practically unchanged. When Zr dopant is added to CeO_2_, a slight decrease in the crystallite size is observed (from 28 nm to up to 26 nm). The simultaneous addition of Mn and Zr dopants to CeO_2_ leads to a significant reduction in the crystallite sizes. It is worth noting the significant reduction in the crystallite size (up to 7 nm) for the Ce_0.5_Mn_0.2_Zr_0.3_O_2_ sample, which is also characterized by the most significant decrease in the ceria lattice parameter.

### 3.4. Raman Spectroscopy

**Figure 4:**
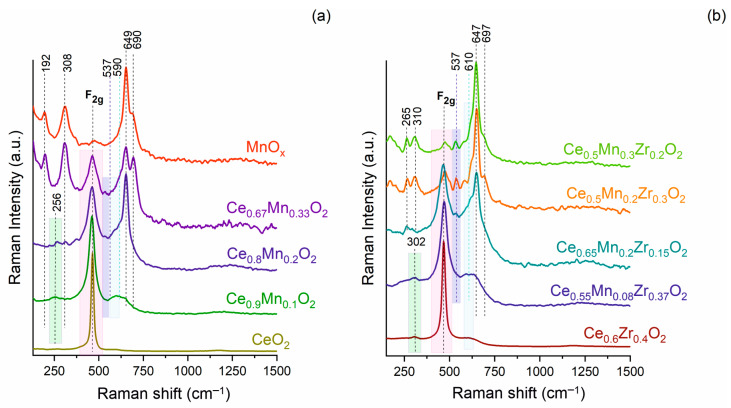
Raman spectra for the prepared samples: (**a**) individual and Ce-Mn oxides, (**b**) Ce-Zr and ternary oxides.

The Raman spectrum for the CeO_2_ sample taken under laser irradiation at 532 nm (Figure 4a) is characterized by a strong Raman band at 464 cm^−1^, which is assigned to the F_2g_ vibrational active mode in the fluorite-type CeO_2_ structure. A weaker band at 1150 cm^−1^ is an overtone of the F_2g_ mode [38]. A band at ~594 cm^−1^ can be assigned to the presence of oxygen vacancies. For the MnO_x_ sample, the vibrations are related to both Mn_3_O_4_ and Mn_2_O_3_ [39].

When Mn is added to CeO_2_, the F_2g_ band position in the spectra for CeMnO_x_ samples slightly shifts towards the lower wavenumber from 464 to 460 cm^−1^, and as the Mn content gradually increases, the F_2g_ band decreases in intensity and becomes broader. The shifts and broadening of the F_2g_ band are connected with the presence of surface oxygen vacancies, a loss of regularity in the crystalline network, and the ∆d/d microstrain effect [40,41,42].

For the Ce_0.9_Mn_0.1_O_2_ sample, the appearance of a broad band at ~256 cm^−1^ is observed, which can be attributed to the displacement of atoms from the ideal positions of the fluorite lattice [43,44]. A broad asymmetric band in the range of 540–718 cm^−1^ can be deconvoluted into the 590 cm^−1^ band attributed to defect areas involving the Mn^n+^ cation in an 8-fold O^2−^ coordination, without any vacancies [45,46], and/or O^2−^ interstitial oxygen defects of the Frenkel type in CeO_2_ [47,48,49,50]. The bands at 649 and 690 cm^−1^ can be attributed to the presence of α-Mn_2_O_3_ [51,52].

For the Ce_0.8_Mn_0.2_O_2_ and Ce_0.67_Mn_0.33_O_2_ samples, the bands at 192, 308, 649, and 690 cm^−1^ become more intense, and they are associated with the Mn–O vibrations in the α-Mn_2_O_3_ [53]. The appearance of weak bands at 310 and 366 cm^−1^ along with a strong band at 649 cm^−1^, which is clearly overlapped with one of the bands related to α-Mn_2_O_3_, is associated with the vibrational stretching of the Mn-O bond towards Mn^2+^ ions in the spinel structure of Mn_3_O_4_ [53]. In addition, the appearance of the band at 537 cm^−1^, which is associated with defect areas including O^2−^ vacancies and is observed when the dopant cations in a M^3+^ state are introduced into the CeO_2_ lattice [45,54], and as mentioned above, the shoulder at 590 cm^−1^, are associated with the defect areas in the 8-fold coordination of O^2−^. Thus, for the CeMnO_x_ samples, the introduction of Mn ions, mainly Mn^3+^, into the CeO_2_ lattice is observed, while the formation of α-Mn_2_O_3_ and Mn_3_O_4_ cannot be excluded, which is consistent with the XRD data.

The spectrum for the binary oxide Ce_0.6_Zr_0.4_O_2_ (Figure 4b) features the F_2g_ band and the broad weak bands at 300 and 612 cm^−1^ associated with the tetragonal substitution of oxygen atoms from the ideal fluorite lattice after the Zr introduction and the formation of oxygen vacancies, respectively [37,55].

For ternary oxide samples, the F_2g_ band is shifted towards the higher wavenumber from 464 to 476 cm^−1^. For ternary samples, the bands related to Mn_3_O_4_ and Mn_2_O_3_ appear. The asymmetry of the band at 649 cm^−1^ and the presence of a band at 310 cm^−1^ may be a consequence of the overlapping of the bands of MnO_x_ species and the presence of oxygen vacancy defects in CeO_2_. A shoulder at 590 cm^−1^ is shifted to ~610 cm^−1^. According to Ref. [45], the band at ~560 cm^−1^ related to the presence of oxygen vacancies can be due to the different oxidation state of the dopant compared to that of Ce^4+^. The band at ~600 cm^−1^ is because of the different ionic radius of the dopant ions compared to that of Ce^4+^. The bands above 650 cm^−1^ can be assigned to the extrinsic defects in ceria as well as aliovalent defects caused by the presence of Mn-related phases where Mn exists in higher oxidation states [56,57]. It is noteworthy that the increase in the Mn + Zr fraction in the series of samples Ce_0.65_Mn_0.2_Zr_0.15_—Ce_0.5_Mn_0.3_Zr_0.2_—Ce_0.5_Mn_0.2_Zr_0.3_ increased the intensity of the bands at 536, 584, and 616 cm^−1^. Thus, the increase in the amount of substituting metals in the CeO_2_ structure results in an increase in the concentration of internal and external defects.

### 3.5. Temperature-Programmed Reduction in Hydrogen (TPR-H_2_)

**Figure 5:**
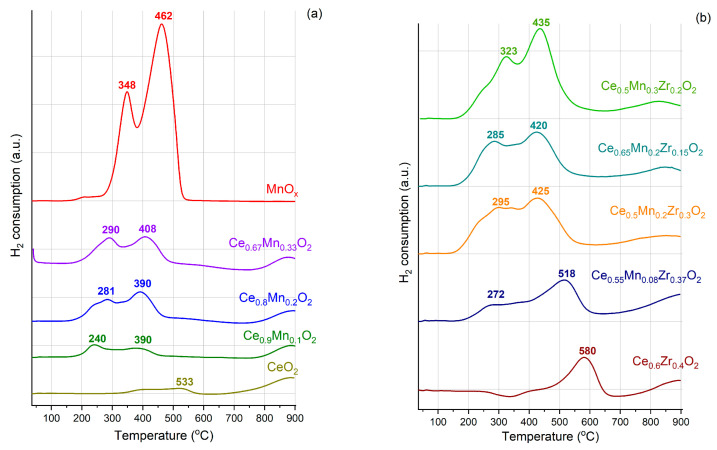
TPR profiles for the obtained samples: (**a**) individual and Ce-Mn oxides, (**b**) Ce-Zr and ternary oxides.

The reduction profile for the MnO_x_ sample is represented by a peak centered at 348 °C (Figure 5), which corresponds to the Mn_2_O_3_ reduction to Mn_3_O_4_, and a peak at 463 °C that corresponds to the Mn_3_O_4_ reduction to MnO [58,59]. The reduction profile for CeO_2_ is characterized by the presence of two temperature regions of hydrogen consumption, namely, the consumption peak in the temperature region of 300–600 °C, at which the surface of ceria nanoparticles is reduced, and at temperatures above 700 °C, at which the bulk of ceria nanoparticles is reduced [60,61]. The main reduction process for ZrO_2_ occurs above 1000 °C (not shown for clarity) [62].

The profiles for the CeMnO_x_ composites are characterized by two regions associated with the reduction of the highly dispersed Mn_2_O_3_ species (200–350 °C) and the reduction of Mn_3_O_4_ particles along with the surface CeO_2_ (350–650 °C) [63,64]. Both peak maxima are shifted progressively to lower temperatures with the increase in the Ce content and a decrease in the Mn content, indicating a progressively stronger interaction between the two oxides. It is noteworthy that the intensity ratio between the two peaks differs when the manganese to cerium content varies. This is due to both different phase ratios of Mn_2_O_3_/Mn_3_O_4_ and the strength of the MnO_x_ interaction with CeO_2_.

The Zr-containing catalysts (Ce_0.6_Zr_0.4_O_2_ and CeMnZrO_x_) are reduced more easily due to the simultaneous reduction of the surface and bulk ceria caused by the introduction of Zr and/or Mn. The progressive substitution of Ce by Zr shifts the maxima towards higher temperatures, i.e., decreases reducibility. This can be because the CeO_2_-ZrO_2_ interaction reduces the CeO_2_-MnO_x_ interactions.

Table 4 presents data on the amount of H_2_ consumed. For binary and ternary oxide systems, there is an increase in the amount of consumed hydrogen, which indicates an increase in the reactivity for these systems. The increase in both total H_2_ consumption (column Σ) and hydrogen consumption at low temperatures (150–500 °C) due to the simultaneous reduction of highly dispersed MnO_x_ particles and the surface of ceria particles indicates a positive effect of both Mn and Zr on reducibility of the CeO_2_ phase.

### 3.6. XPS

**Figure 6:**
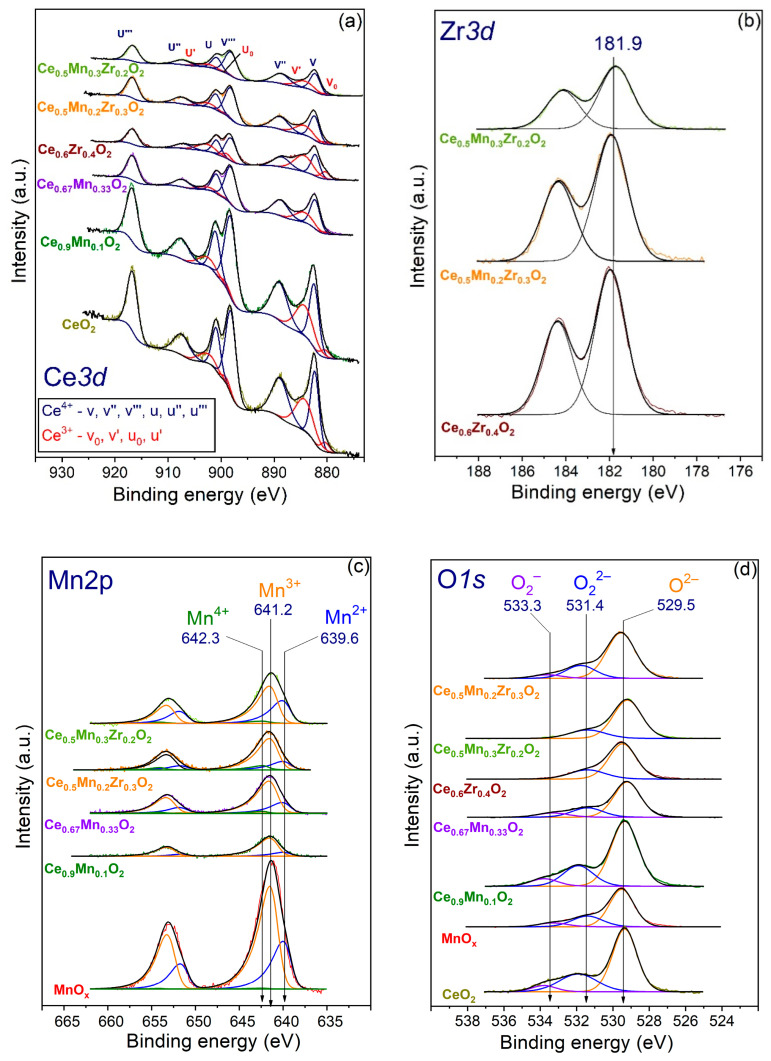
The core-level XPS spectra for individual, binary, and ternary oxide in the CeO_2_-ZrO_2_-MnO_x_ system: (**a**) Ce3d; (**b**) Zr3d; (**c**) Mn2p; (**d**) O1s.

To study the states of elements on the sample surfaces, the XPS method was employed. Figure 6 and Table 5 show the XPS Ce3d, Mn2p, Zr3d, and O1s spectra and the relative concentrations (atomic ratios) of elements in the subsurface layer, respectively. The normalization of spectra to the total integral intensity (for the Ce3d, Mn2p, and Zr3d spectra) of the corresponding spectra for supports was carried out. The analysis of the spectra is based on their deconvolution.

The XPS Ce3d spectrum (Figure 6a) shows the presence of Ce^3+^ and Ce^4+^ states, and the splitting into sublevels Ce3d_5/2_ and Ce3d_3/2_ occurs due to the spin–orbit interaction. Three lines at v/u, v″/u″, and v‴/u‴ and two lines at v′/u′ and v_0_/u_0_ are related to CeO_2_ and Ce_2_O_3_, respectively [41]. The Ce^3+^ and Ce^4+^ states are characterized by the peaks at v_0_, v′, u_0_, and v′ and v, v″, v‴, u, u″, and u‴, respectively. The Zr3d spectra (Figure 6b) feature the Zr3d_5/2_ binding energy of 181.9 eV related to the Zr^4+^ state. The Mn2p spectra (Figure 6c) contain Mn2p_3/2_–Mn2p_1/2_ doublets, where peaks at 639.7, 641.2, and 642.2 eV correspond to Mn^2+^, Mn^3+^, and Mn^4+^ states, respectively [65] that can be attributed to MnO, Mn_2_O_3_, and MnO_2_ [66,67], respectively. The deconvolution of O1s spectra (Figure 6d) contain three peaks with the binding energies of 533.8, 531.7, and 529.4 eV. These peaks are attributed to O_2_^−^, O_2_^2−^, and O^2−^ states that correspond to adsorbed water, active surface O species/surface hydroxyls, and lattice oxygen [68].

The Ce^3+^ fraction increases as the Mn content decreases in the series of samples MnO_x_—Ce_0.67_Mn_0.33_O_2_—Ce_0.5_Mn_0.3_Zr_0.2_O_2_—Ce_0.5_Mn_0.2_Zr_0.3_O_2_—Ce_0.9_Mn_0.1_O_2_—CeO_2_. The Ce_0.67_Mn_0.33_O_2_ sample features the lowest value. The samples feature rather low fractions of the Mn^4+^ state, the fraction of the Mn^3+^ state increases and the one of the Mn^2+^ state decreases, with the highest Mn^3+^ and lowest Mn^2+^ fractions being reached over Ce_0.9_Mn_0.1_O_2_, which also features the highest fraction of active oxygen. The lowest fraction of active oxygen is shown by the Ce_0.5_Mn_0.3_Zr_0.2_O_2_ sample. A higher fraction of the Mn^3+^ state in the samples is usually related to a lower fraction of the O^2−^ state.

### 3.7. TEM

**Figure 7:**
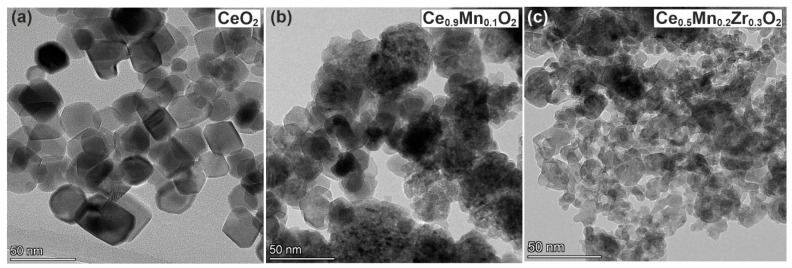
TEM images of (**a**) CeO_2_; (**b**) Ce_0.9_Mn_0.1_O_2_; (**c**) Ce_0.5_Mn_0.3_Zr_0.2_O_2._

Figure 7 shows the TEM images for the synthesized CeO_2_, binary, and ternary oxides. The CeO_2_ sample is characterized by the presence of well-crystallized particles with a size of 10–40 nm, which is consistent with the XRD data. Such a structure is caused by the hydrothermal treatment of the oxide during the preparation. The structure of binary Ce_0.9_Mn_0.1_O_2_ oxide is significantly different from the one of the CeO_2_ sample. Loose aggregates of particles with the size of a few nanometers are observed. Thus, the addition of even small amounts of Mn into the CeO_2_ leads to significant changes in the sample structure. The ternary Ce_0.5_Mn_0.3_Zr_0.2_O_2_ oxide is also characterized by the presence of loose aggregates of small particles. The TEM images show that the surface of the samples is determined by the surface of the primary particles, and the growth of the specific surface area for binary and ternary oxides is a result of the decrease in the particle size. Additionally, the porous structure of the sample is represented by the interparticle space and the mesoporous structure of the sample can be well observed in the presented TEM images.

**Figure 8:**
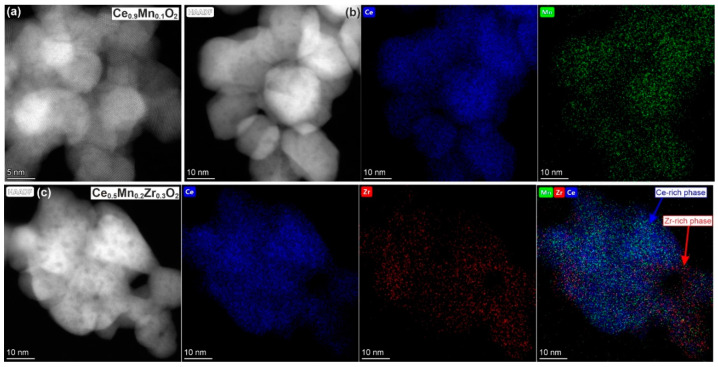
STEM-HR image for (**a**) Ce_0.9_Mn_0.1_O_2_ sample. HAADF-STEM and Ce and Mn maps for (**b**) Ce_0.9_Mn_0.1_O_2_; (**c**) Ce_0.5_Mn_0.3_Zr_0.2_O_2._

To determine the distribution of elements in the binary and ternary oxides, HR TEM and EDX analysis were applied. For both Ce_0.9_Mn_0.1_O_2_ and Ce_0.5_Mn_0.3_Zr_0.2_O_2_, only the CeO_2_ phase is determined, while the interlayer distances for the ZrO_2_ or MnO_x_ phases are not identified. Figure 8a shows the HR-STEM image for the Ce_0.9_Mn_0.1_O_2_ sample, and well-crystallized particles with sizes of 4–10 nm and the interlayer distance of the CeO_2_ phase may be observed. The maps of Ce and Mn distribution (Figure 8b) show the uniform Mn distribution in the sample. Mn can be stabilized as highly dispersed species on the CeO_2_ surface and/or as Mn ions incorporated into the CeO_2_ structure. Separated MnO_x_ particles are not observed, which is consistent with the XRD data.

Figure 8c shows the distribution of metals for ternary Ce_0.5_Mn_0.3_Zr_0.2_O_2_ oxide. The Ce and Zr distributions demonstrate that the Ce-rich and Zr-rich areas can be highlighted. This is consistent with the XRD results because two Ce_1−x_Zr_x_O_2_ and Ce_x_Zr_1−x_O_2_ phases are observed for the Ce_0.6_Zr_0.4_O_2_ sample and ternary CeO_2_-ZrO_2_-MnO_x_ samples. The Ce-rich phase is predominant, and it is also consistent with the XRD data. The Mn distribution is relatively uniform, and the stabilization of the MnO_x_ clusters on the surface and its incorporation into both Ce-rich and Zr-rich mixed oxide cannot be excluded. The Mn-related species seem to be localized mostly near the areas rich in ceria and less near the Zr-rich areas.

### 3.8. Catalytic Activity in CO and Soot Oxidation

**Figure 9:**
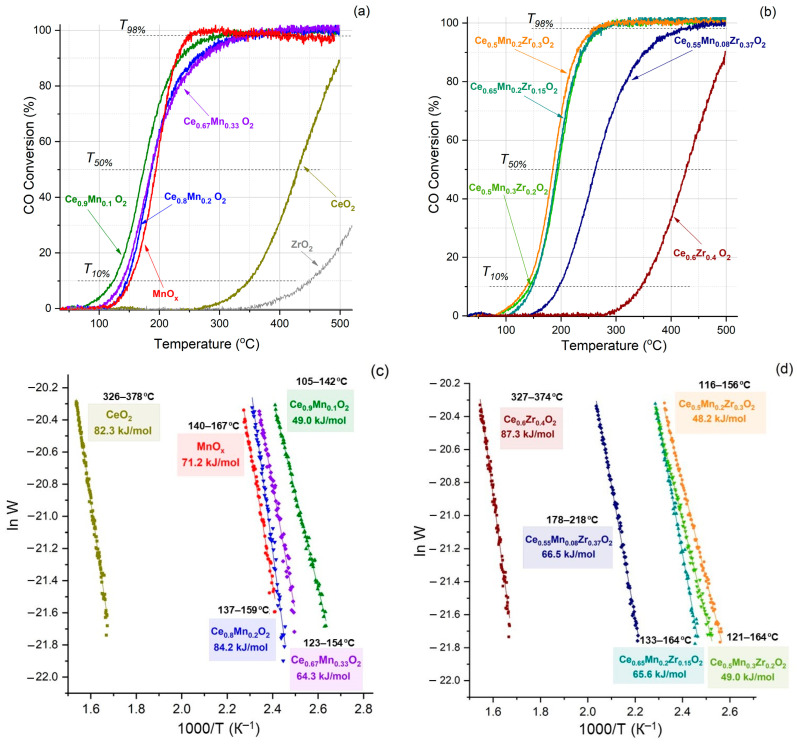
Catalytic properties of prepared systems in CO oxidation: (**a**) individual and Ce-Mn oxides, (**b**) Ce-Zr and ternary oxides; (**c**,**d**) corresponding activation energies.

The catalytic activity of the obtained samples in CO oxidation was studied (Figure 9). For all samples, with the exception of cerium and zirconium oxides, 100% conversion was achieved at temperatures below 450 °C. Binary systems comprising cerium and manganese oxides are characterized by the onset of conversion at ~100 °C. As the manganese content increases in these samples, a decrease in the catalytic activity is observed. The most active is Ce_0.9_Mn_0.1_O_2_, for which 50% and 98% conversion is achieved at temperatures of 174 °C and 298 °C, respectively, with an activation energy of 49.0 kJ/mol (Figure 9c). Ternary oxide systems typically begin conversion also at ~100 °C. The Ce_0.55_Mn_0.08_Zr_0.37_O_2_ sample is the least active: 50% and 98% conversions are achieved at 261 °C and 414 °C, respectively. The Ce_0.5_Mn_0.2_Zr_0.3_O_2_ sample shows the highest activity: T_50%_ and T_98%_ are 184 °C and 260 °C, respectively, and the activation energy is 48.2 kJ/mol (Figure 9d).

**Figure 10:**
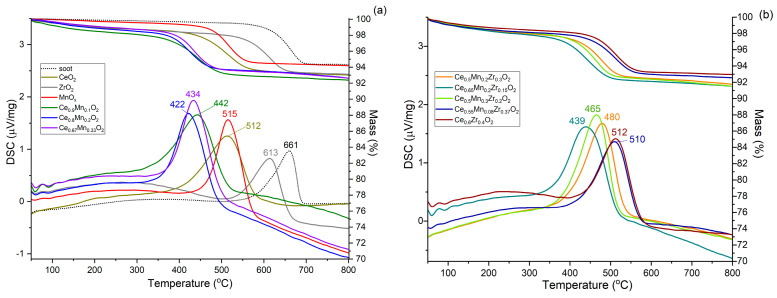
Soot combustion activity of prepared samples: (**a**) individual and Ce-Mn oxides, (**b**) Ce-Zr and ternary oxides.

Simultaneous thermal analysis was used to determine the catalytic activity of oxide catalysts in soot oxidation. Figure 10 shows the TG and DSC curves obtained from soot oxidation simulations using the resulting catalysts. Non-catalytic soot oxidation is observed in the temperature range 550–700 °C with T_max_ at 661 °C. The presence of ZrO_2_ leads to a slight increase in soot oxidation activity (T_max_ = 613 °C) (not shown). The presence of cerium and manganese oxides decreases the maximum temperature to 512 °C and 515 °C, respectively. The presence of binary and ternary oxide systems shifts the reaction temperature range to 350–600 °C (i.e., it decreases the reaction temperature range by 100–170 °C). Samples Ce_0.8_Mn_0.2_O_2_ and Ce_0.65_Mn_0.2_Zr_0.15_O_2_ showed the highest activity: the maximum conversion temperatures for these samples were 422 and 439 °C, respectively. Comparisons of soot combustion and CO oxidation performances of the prepared samples and counterparts presented in the literature are presented in Tables S1 and S2 (Supplementary materials). The materials obtained show comparable activity.

## 4. Discussion

The results of the catalytic studies show that the binary Ce-Mn catalysts demonstrate higher performance in both CO oxidation (Ce_0.9_Mn_0.1_O_2_) and soot combustion (Ce_0.8_Mn_0.2_O_2_) reactions, which are closely followed by the ternary oxide catalysts. The key features of the Ce_0.9_Mn_0.1_O_2_ sample, which also shows a rather low T_max_ value in soot combustion, include the following: (1) the presence of both small (2–4 nm) and large (5–50 nm) pores; (2) the formation of Ce_0.94_Mn_0.06_O_2−δ_ solid solution where the Mn component can both be included into the ceria structure and form several highly dispersed surface species related to Mn oxides; (3) the lowest value of H_2_ consumption in the temperature range of 150–500 °C (related to the reduction of dispersed MnO_x_ and surface CeO_2_) and the highest one at temperatures above 650 °C (related to bulk CeO_2_); (4) the highest fractions of Mn^3+^ state and active oxygen; and (5) the predominant presence of intrinsic and Frenkel-type oxygen vacancies. Sample Ce_0.8_Mn_0.2_O_2_ features include the following: (1) high pore volume (0.290 cm^3^/g, the largest in the whole series) and large pore sizes (24.4 nm), with the latter being predominantly formed; (2) the presence of the solid solution with the estimated composition of Ce_0.9_Mn_0.1_O_2−δ_ where the content of highly dispersed surface Mn oxides species is even higher as compared to Ce_0.9_Mn_0.1_O_2_; (3) higher impact of microstrains as compared to Ce_0.9_Mn_0.1_O_2_; and (4) higher concentration of extrinsic MnO_x_-related defects (Raman spectrum). The interplays of the mentioned features can be the reasons for the observed activity.

The ternary Ce_0.5_Mn_0.2_Zr_0.3_O_2_ features a higher specific surface and narrower particle size distribution as compared to the mentioned binary samples. The sample shows high H_2_ consumption in the temperature ranges below 500 °C and above 650 °C. The sample is characterized by a higher fraction of high oxidation states of Mn (i.e., 3+, 4+). The formation of solid solutions with different compositions, say, Ce_1−x_Mn_x_O_2_ and Ce_1−x_Zr_x_O_2_, and their interplay on the surface can also be the reason for the observed performance.

The mentioned features imply that the surface of the binary Ce-Mn samples with low Mn content is represented by a “cocktail” of active sites (e.g., oxygen vacancies, highly dispersed MnO_x_ species with various compositions, CeO_2_-MnO_x_ interfaces, etc.) that coexist, acting separately and/or synergistically and allowing the samples to exhibit high activity in conversion of both CO and soot. The observed differences in performance can be attributed to the effect of Mn loading, where lower Mn content ensures the formation of a balanced mixture of MnO_x_ species, while higher Mn content probably results in the formation of the Mn-related species that partially block other active sites to reduce overall performance. The formation of interfaces between various Mn-related species that are less catalytically active in said reactions cannot be excluded.

## 5. Conclusions

In the present work, a series of CTAB-templated CeO_2_-ZrO_2_-MnO_x_ catalysts was prepared by coprecipitation of the corresponding precursors. The addition of Mn and/or Zr dopant to ceria improved its performance in both CO oxidation and soot combustion. A complex of physical–chemical methods (low-temperature N_2_ sorption, XRD, TPR-H_2_, Raman, HR TEM, XPS) allowed revealing the structural and compositional peculiarities of the catalysts that caused the observed performance. Binary Ce-Mn catalysts demonstrated higher performance, and were closely followed by the ternary oxide catalysts. The reason was linked with the formation of several types of active sites, namely, highly dispersed MnO_x_ species, interfaces, and oxygen vacancies that can act individually and/or synergistically to allow said samples to be active in both reactions. Future work can include revealing the intrinsic activity and mechanisms of action of such species in environmental catalytic reactions.

## Figures and Tables

**Table 1:** Chemical composition (XRF) (*) and Ce/Zr, Ce/Mn, and Zr/Mn ratios (XRF and XPS).

Sample	n(Ce)/ n(Sum)	n(Mn)/ n(Sum)	n(Zr)/ n(Sum)	Ce/Zr		Ce/Mn		Zr/Mn	
				XRF	XPS	XRF	XPS	XRF	XPS
Ce_0.9_Mn_0.1_O_2_	0.915	0.085	–	–	–	10.76	9.20	–	–
Ce_0.8_Mn_0.2_O_2_	0.738	0.262	–	–	–	2.82	–	–	–
Ce_0.67_Mn_0.33_O_2_	0.686	0.314	–	–	–	2.18	2.41	–	–
Ce_0.6_Zr_0.4_O_2_	0.648	–	0.351	2.06	1.43	–	–	–	–
Ce_0.5_Mn_0.2_Zr_0.3_O_2_	0.544	0.184	0.272	2.00	0.93	3.09	1.59	1.48	1.71
Ce_0.65_Mn_0.2_Zr_0.15_O_2_	0.692	0.176	0.132	5.24	n.d.	3.93	n.d.	0.75	n.d.
Ce_0.5_Mn_0.3_Zr_0.2_O_2_	0.459	0.382	0.158	2.90	2.98	1.20	1.37	0.41	0.46
Ce_0.55_Mn_0.08_Zr_0.37_O_2_	0.609	0.045	0.346	1.76	n.d.	13.53	n.d.	7.69	n.d.

(*) expressed as atomic ratio of metal to total of metals (sum).

**Table 2:** Textural properties of samples according to low-temperature nitrogen adsorption data.

Sample	SSA, m^2^/g	V, cm^3^/g	Pore Size, nm
MnO_x_	6	0.03	37
ZrO_2_	39	0.11	8
CeO_2_	37	0.18	19
Ce_0.9_Mn_0.1_O_2_	40	0.18	19
Ce_0.8_Mn_0.2_O_2_	45	0.29	24
Ce_0.67_Mn_0.33_O_2_	38	0.26	27
Ce_0.6_Zr_0.4_O_2_	46	0.11	8
Ce_0.5_Mn_0.2_Zr_0.3_O_2_	52	0.16	10
Ce_0.5_Mn_0.3_Zr_0.2_O_2_	46	0.18	14
Ce_0.55_Mn_0.08_Zr_0.37_O_2_	50	0.13	8
Ce_0.65_Mn_0.2_Zr_0.15_O_2_	52	0.24	17

**Table 3:** Phase composition of samples and structural parameters, microstrains, and crystallite sizes of the crystalline phases identified from XRD data.

Sample	Phase	Structural Parameters				
		*a*, Å	*b, Å*	*c*, Å	∆d/d × 10^−3^	D_XRD_, nm
CeO_2_	cub. CeO_2_	5.407	-	-	0.99	28
MnO_x_	cub. Mn_2_O_3_	9.404	-	-	0.91	43
ZrO_2_	mon. ZrO_2_	5.147	5.2032	5.313	3.69	15
	tetr. ZrO_2_	3.597	-	5.177	1.92	15
Ce_0.9_Mn_0.1_O_2_	cub. Ce_1−x_Mn_x_O_2−δ_	5.387	-	-	2.16	23
Ce_0.8_Mn_0.2_O_2_	cub. Ce_1−x_Mn_x_O_2−δ_	5.369	-	-	3.76	24
Ce_0.67_Mn_0.33_O_2_	cub. Ce_1−x_Mn_x_O_2−δ_	5.366	-	-	5.31	24
Ce_0.6_Zr_0.4_O_2_	cub. Ce_1−x_Zr_x_O_2−δ_	5.393	-	-	7.32	26
	cub. Ce_x_Zr_1−x_O_2−δ_/ tetr. Ce_x_Zr_1−x_O_2−δ_	5.229/ 3.624	-	-/ 5.277	1.36	8
Ce_0.5_Mn_0.2_Zr_0.3_O_2_	cub. Ce_1−x_Zr_x_O_2−δ_	5.331	-	-	5.90	7
	tetr. Ce_x_Zr_1−x_O_2−δ_	3.701	-	5.339	9.2	5
	cub. Mn_2_O_3_	n.a.	-	-	n.a.	n.a.
Ce_0.65_Mn_0.2_Zr_0.15_O_2_	cub. Ce_1−x_Zr_x_O_2−δ_	5.343	-	-	4.35	17
	cub. Mn_2_O_3_	n.a.	-	-	n.a.	n.a.
Ce_0.5_Mn_0.3_Zr_0.2_O_2_	cub. Ce_1−x_Zr_x_O_2−δ_	5.347	-	-	9.38	21
	tetr. Ce_x_Zr_1−x_O_2−δ_	3.696	-	5.277	9.4	4
	cub. Mn_2_O_3_	n.a.	-	-	n.a.	n.a.
Ce_0.55_Mn_0.08_Zr_0.37_O_2_	cub. Ce_1−x_Zr_x_O_2−δ_	5.354	-	-	7.56	20
	tetr. Ce_x_Zr_1−x_O_2−δ_	3.696	-	5.277	4.2	7

**Table 4:** The H_2_ consumption in TPR for the prepared catalysts.

**Sample**	**H_2_ Consumption (mmol/mol Catalyst)**			
	**150–350 °C**	**350–650 °C**	**600–900 °C**	**Σ**
CeO_2_	-	0.024 (surface CeO_2_)	0.057 (bulk CeO_2_)	0.081
MnO_x_	0.215 (dispersed MnO_x_)	0.472 (reduction of MnO_x_)	-	0.687
Ce_0.6_Zr_0.4_O_2_	-	0.031	0.016	0.047
**Catalysts**	**150–500 °C (Dispersed MnO_x_ and Surface CeO_2_)**	**500–650 °C (Surface CeO_2_)**	**650–900 °C** **(Bulk CeO_2_)**	**Σ**
Ce_0.9_Mn_0.1_O_2_	0.060	0.002	0.038	0.669
Ce_0.8_Mn_0.2_O_2_	0.130	0.001	0.037	1.296
Ce_0.67_Mn_0.33_O_2_	0.146	0.001	0.032	1.426
Ce_0.5_Mn_0.2_Zr_0.3_O_2_	0.120	0.005	0.027	1.233
Ce_0.65_Mn_0.2_Zr_0.15_O_2_	0.110	0.003	0.015	1.112
Ce_0.5_Mn_0.3_Zr_0.2_O_2_	0.173	0.015	0.033	1.702
Ce_0.55_Mn_0.08_Zr_0.37_O_2_	0.065	0.020	0.025	0.675

**Table 5:** Relative concentrations of elements in the near-surface layer of the samples.

Sample	Ce^4+^, %	Mn^2+^, %	Mn^3+^, %	Mn^4+^, %	(O_2_^2−^ + O_2_^−^)/O_t_^1^	O^2−^, %	O_2_^2−^, %	O_2_^−^, %
MnO_x_	–	31	69	1	0.32	68	24	8
Ce_0.67_Mn_0.33_O_2_	85	24	76	4	0.31	69	21	10
Ce_0.5_Mn_0.3_Zr_0.2_O_2_	84	38	62	5	0.22	78	22	0
Ce_0.5_Mn_0.2_Zr_0.3_O_2_	82	19	73	9	0.27	73	21	6
Ce_0.9_Mn_0.1_O_2_	81	17	83	n.d.	0.32	68	24	8
Ce_0.6_Zr_0.4_O_2_	65	–	–	–	0.25	75	25	0
CeO_2_	80	–	–	–	0.35	65	29	6

^1^ O_t_ = O_2_^2−^ + O_2_^−^ + O^2−^.

## Data Availability

Data are contained within the article and Supplementary materials.

## Supplementary Materials

The following supporting information can be downloaded at: https://www.mdpi.com/article/10.3390/nano13243108/s1. Table S1. A comparison of soot combustion activity for the prepared samples and a selection of counterparts. Table S2. A comparison of CO oxidation activity for the prepared samples and a selection of counterparts. References [69,70,71,72] were also cited in the supplementary materials.
