# Supplementary material for: Unraveling the Structural and Compositional Peculiarities in CTAB-Templated CeO2-ZrO2-MnOx Catalysts for Soot and CO Oxidation
Source: Nanomaterials (Basel). 2023 Dec 9;13(24):3108. doi: 10.3390/nano13243108 (PMC10745992; doi:10.3390/nano13243108)
Supplement: Supplementary file 1 [file nanomaterials-13-03108-s001.zip › nanomaterials-2739588-supplementary.pdf]

Table S1. A comparison of soot combustion activity for the prepared samples and a selection of counterparts.

Catalyst composition	Preparation method	Catalyst/soot ratio	Contact mode	Reaction conditions	T_{max}, °C	Ref.
$Ce_{0.8}Mn_{0.2}O_2$	CTAB-templated	1/20	TC	Air (80%), Ar balance total flow=100 mL.min^{-1}	422	This work
$Ce_{0.65}Mn_{0.2}Zr_{0.15}O_2$					439	
$Ce_{0.9}Mn_{0.1}O_2$	Citrate sol-gel	1/20	TC	Air (80%), Ar balance total flow=100 mL.min^{-1}	476	[1]
$Ce_{0.5}Mn_{0.2}Zr_{0.3}O_2$					490	
$Mn_{10}Ce_{90}$(1-CA)	citric acid-assisted method	1/20	TC	Air flow	387 ($T_{50\%}$)	[2]
		1/20				
Soot	co-precipitation method	1/10	TC	$[O_2]$ = 0.5%, N_2 balance flow rate = 500 mL/min	~670	[3]
CZ			TC		~390	
M10-CZ			TC		~370	
M15-CZ			TC		~375	
M			TC		~410	
CZ			LC		~615	
M10-CZ			LC		~610	
M15-CZ			LC		~610	
M			LC		~670	

Table S2. A comparison of CO oxidation activity for the prepared samples and a selection of counterparts.

Catalyst composition	Preparation method	Conditions	T_{50}, °C	T_{100}, °C	Ea, kJ/mol	Ref.
$Ce_{0.9}Mn_{0.1}O_2$	CTAB-templated	1 vol% CO, 5 vol% O_2, and 94 vol% He, 0.1 g, flow rate=100 mL/min, WHSV=1000 mL/min•g	~170	~280	49	This work
$Ce_{0.5}Mn_{0.2}Zr_{0.3}O_2$			~184	~260	48	
$Ce_{0.9}Mn_{0.1}O_2$	Citrate sol-gel	1 vol% CO, 5 vol% O_2, and 94 vol% He, 0.1 g, flow rate=100 mL/min, WHSV=1000 mL/min•g	188	235	70	[1]
$Ce_{0.5}Mn_{0.2}Zr_{0.3}O_2$			148	180	50	
Mn-CZ (MnO_x-$Ce_{0.8}Zr_{0.2}O_2$)	EISA method using CTAB template	2 vol% CO and 1 vol% O_2 in He 0.1 g, flow rate=60 mL/min, WHSV=600 mL/min•g	~164	280	N/A	[4]

MnO_x-ZrO_2	co-precipitation	1 vol.% CO in air<br>0.5 g, flow rate=487 mL/min<br>WHSV=974 mL/min•g	~180	450	N/A	[[5]]
Ag/CeO_2-600 (4.65 wt.% Ag)	PLA	0,2% CO, 1,0 % O2<br>240 000 ч-1<br>1000 мл/мин, 0,2 г	210	300	N/A	[[6]]

---

[1] Grabchenko, M.V.; Mamontov, G.V.; Chernykh, M.V.; Vodyankina, O.V.; Salaev, M.A. Synergistic effect in ternary CeO_2-ZrO_2-MnO_x catalysts for CO oxidation and soot combustion. Chemical Engineering Science. Accepted.

[2] Sacco N.A.; Bortolozzi J.P.; Milt V.G.; Miró E.E.; Banús E.D. One step citric acid-assisted synthesis of Mn-Ce mixed oxides and their application to diesel soot combustion, *Fuel*, 2022, 124201. https://doi.org/10.1016/j.fuel.2022.124201.

[3] Yao, P.; He, J.; Jiang, X.; Jiao, Y.; Wang, J.; Chen, Y. Factors determining gasoline soot abatement over CeO_2–ZrO_2-MnO_x catalysts under low oxygen concentration condition, *J. Energy Inst.*, 2020, 93, pp. 774-783. https://doi.org/10.1016/j.joei.2019.05.005.

[4] Kaplin, I.Y.; Lokteva, Ekaterina, S.; Golubina, Elena V.; Shishova, Vera V.; Maslakov, Konstantin I.; Fionov, Alexander V.; Isaikina, Oksana Ya.; Lunin, Valery V. Efficiency of manganese modified CTAB-templated ceria-zirconia catalysts in total CO oxidation. *Applied Surface Science.* 2019, Volume 485, pp. 432 – 440. DOI: 10.1016/j.apsusc.2019.04.206.

[5] Bulavchenko, O.A.; Konovalova, V.P.; Saraev, A.A.; Kremneva, A.M.; Rogov, V.A.; Gerasimov, E.Y.; Afonasenko, T.N. The Catalytic Performance of CO Oxidation over MnO_x-ZrO_2 Catalysts: The Role of Synthetic Routes, *Catalysts*, 2023, volume 13, 57. https://doi.org/10.3390/catal13010057.

[6] Kibis L.S. Interface Interactions And CO Oxidation Activity Of Ag/CeO_2 Catalysts: A New Approach Using Model Catalytic Systems, *Appl. Catal. A: Gen.*, 2019, Vol. 570. pp. 51-61.
